# Physicians’ experiences of assessing and supporting fatigued patients in primary care: a focus group study

**DOI:** 10.1186/s12875-025-02891-1

**Published:** 2025-06-09

**Authors:** Conrad Samuelsson, Lisa Gunnarsson, Frank Svärdman, Christian Rück, Elin Lindsäter

**Affiliations:** 1 Gustavsberg University Primary Healthcare Clinic, Academic Primary Care Center, Region Stockholm, Stockholm, Sweden; 2 Division of Psychology, Department of Clinical Neuroscience, Karolinska Institutet, Stockholm, Sweden; 3 Centre for Psychiatry Research, Department of Clinical Neuroscience, Karolinska Institutet & Stockholm Health Care Services, Region Stockholm, Stockholm, Sweden

**Keywords:** Primary Healthcare, Fatigue, Focus Groups, Health Care Process Assessment

## Abstract

**Background:**

Fatigue is a common symptom in primary healthcare, affecting 10–30% of patients, and is associated with increased healthcare consumption and functional disability. There is a lack of standardised guidelines to assess and treat patients with fatigue, and little is known about how patients are currently managed in primary healthcare. This study aimed to explore physicians' experiences of managing patients with fatigue in Swedish primary care to inform development of evidence-based care procedures.

**Method:**

Six semi-structured focus group interviews were conducted, including a total of 39 primary care physicians from three primary care centres in Stockholm. Data was analysed using thematic analysis.

**Results:**

The analysis generated the overarching theme "Frustration in the role as physician," consisting of two main themes: (1) “Time pressure and an empty toolbox”, highlighting the perceived lack of standardised assessment procedures, effective interventions, and sufficient time for care; and (2) “Challenges in the patient-physician relationship”, highlighting role ambivalence, ambivalence regarding sick leave, and the importance of reaching mutual understanding with the patient.

**Conclusion:**

Physicians often feel frustrated, ill-equipped, and time-pressured when managing patients with fatigue. There is a pressing need to develop evidence-based assessment procedures and treatments in the primary care context.

**Supplementary Information:**

The online version contains supplementary material available at 10.1186/s12875-025-02891-1.

## Background

Fatigue, a feeling of severe, persistent tiredness not alleviated by rest, is reported in 20–30% of all primary care patients and is the primary complaint of up to 10% of patients [1]. Fatigue is a severely debilitating symptom, associated with increased healthcare consumption, work disability, and mortality [2], and is common across a wide range of conditions, including respiratory, cardiovascular, endocrine, gastrointestinal, hematologic, infectious, neurologic, and musculoskeletal diseases, as well as cancer [3, 4]. It is also associated with alcohol dependence, sleep problems, prolonged stress and psychiatric conditions such as depression and post-traumatic stress disorder [3–5]. Among primary care patients presenting with fatigue as their primary complaint, only a minority are diagnosed with a specific disorder that explains the fatigue [6].

In Sweden, the diagnosis “exhaustion disorder” was added to the Swedish version of the International Classification of Diseases (ICD-10-SE) in 2005. Its introduction aimed to establish standardised diagnostic criteria, enable eligibility for sickness absence benefits, and to facilitate research into evidence-based care and rehabilitation strategies [7]. According to its diagnostic criteria, exhaustion disorder is defined as developing from long-standing sub-traumatic life stressors. The clinical picture is primarily characterised by physical and mental fatigue, together with a range of other symptoms such as concentration and memory problems, sleep and mood disturbances, and physical symptoms such as sensitivity to stimuli, gastrointestinal problems, dizziness, nausea, and pain [8]. Since its introduction, the prevalence of exhaustion disorder has increased markedly and currently accounts for 15% of all mental health diagnoses in Swedish primary care [9]. It has also become the single most common cause of long-term sickness absence in the country [10]. As with other conditions where fatigue is a prominent symptom, there are substantial knowledge gaps, few guidelines for healthcare workers, and a lack of evidence-based treatments for exhaustion disorder [11, 12].

Due to its non-specific nature, persistent fatigue often presents a significant challenge to physicians. Qualitative studies of physicians’ perspectives on patients with persistent fatigue in specialised treatment settings indicate that that physicians often have negative associations to fatigue and express difficulties in the clinician-patient relationship [13, 14]. Studies in the primary care context are less numerous but indicate similar frustration [15, 16]. Although multi-factorial biopsychosocial models have emerged as viable options to guide assessment and interventions for fatigue and other persistent physical symptoms in the past decades [4], it remains unknown to what extent such models currently inform Swedish primary care practice.

The aim of the present study is to explore the experiences of primary care physicians when it comes to managing patients with persistent fatigue when no clear and/or treatable somatic cause can be identified. Sweden is unique in its widespread use of the exhaustion disorder diagnosis, but knowledge about how physicians manage the diagnosis in relation to other fatigue-dominated conditions is limited. The study is a step towards identifying areas for improved management of persistent fatigue in primary care.

## Method

### Study design

This study analysed interview data from focus groups of physicians working at three large primary care centres (PCC) in Region Stockholm, Sweden. Six focus groups, two at each PCC, consisting of 39 primary care physicians, were conducted between October 26 th, 2023, and November 21 st, 2023. Focus groups are semi-structured group interviews that are led by a moderator. The method enables participants to react to each other’s statements, generating data pertaining to group attitudes, views, and vocabulary as well as those of individuals [17, 18].

Data was analysed using thematic analysis (TA) [18–20]. TA is a commonly used method in qualitative studies due to its theoretical flexibility and has been described as particularly practical in healthcare research when deep contextual understanding needs to be generated by researchers who are not specialised in qualitative research [21].

Ethical approval was sought for the study in July 2023 and the Swedish Ethical Review Authority determined that no ethical approval was necessary because the study did not collect sensitive personal information (ERB no. 2023–04599-01). The study was pre-registered on Open Science Framework (DOI: 10.17605/OSF.IO/C9YBQ). Consolidated criteria for reporting qualitative research (COREQ) were applied [22].

#### Researcher background and study conception

The main focus of the research group is to develop scalable psychological treatments for primary care patients suffering from persistent fatigue, including exhaustion disorder. CS and EL developed the research questions and the interview protocol based on observations that physicians often express insecurity regarding assessment and management of fatigue. The questions in the interview protocol aimed to probe physician experiences and perceived challenges when it comes to assessing and managing patients with persistent fatigue by asking open-ended questions. Of interest was also how physicians communicate about fatigue with patients, including what diagnostic labels they use and how they discuss inconclusive test results. The semi-structured interview protocol is available in the online supplement.

#### Recruitment procedure

The three PCCs were selected due to being large clinics with many (**≥ **30) employed physicians, and because relations were already established through previous collaborations. The PCCs are publicly funded and cover a wide patient demographic as regards ethnicity, age distribution, employment status, and income. The only eligibility criterium was that the physician had at least one month (full-time equivalent) experience of working in primary care. Department heads were excluded from participating to facilitate openness in the groups. Due to the busy work schedules of physicians in primary healthcare, sample selection was pragmatic. Physicians were informed about the study at their regular team meetings, and were offered a set of possible dates, suggested by the department head. Those who were available and showed interest were included. Interviews were scheduled during lunch to interfere minimally with daily schedule, and the physicians were offered free lunch for their participation. Department heads were asked to inform physicians who could not attend the staff meetings about the study. In total, an estimated 93 eligible physicians were informed of the study, and 40 signed up to participate.

#### Focus group procedure

Focus groups were moderated by the first author (CS), a male psychologist. The second author (LG), a female research assistant, was present at each interview to assist the moderator and monitor group interaction.

Focus groups were held in conference rooms at each PCC and lasted between 35 and 50 min. As participants entered the room, they were given a short survey with questions about their background and experiences of managing fatigued patients (see online supplement). To initiate the interview, all participants were asked to introduce themselves and give their first association to “persistent tiredness or fatigue” as an icebreaker [23].

#### Analysis plan

All focus group interviews were audio-recorded and transcribed verbatim by CS and LG in a common word processor, complemented with the observer notes in the margins. The names of the participants were censored in the transcripts.

The analysis adhered to TA guidelines by Braun and Clarke [18–20]. TA follows a six-step iterative process. (1) Familiarisation: Researchers first read and re-read the transcribed material, including observer notes, to familiarise themselves with all aspects of the data. (2) Initial codes: During this familiarisation, initial ideas for codes and themes were written down by each researcher independently. The researchers then met to discuss ideas and begin coding. The coding process consisted of identifying meaning-bearing text extracts from the data which relate in some way to the research questions, then generating code labels which capture the essential meaning of the extract. All researchers initially coded one interview and then compared their coding during a second meeting to reach consensus about how to code the material. The full material was subsequently coded by CS. Codes were organised into a codebook using spreadsheet software where recurrences of codes across the material could easily be referenced. Codes were then reviewed by CS and LG and clustered to reduce redundancies. (3) Searching for themes: The remaining codes were organised into themes by CS and reviewed against the codebook and against the entire data set. (4) Reviewing themes: This process was iterative, with new theme conceptualisations formulated as a result of reviewing the data through the lens of the new themes. During this period, new theme conceptualisations were discussed with the entire research group in joint meetings, with the process continuing until the research team was satisfied that the themes captured the shared interpretation of the data. (5) Refining, defining, and naming themes was done in collaboration with all researchers alongside (6) the writing of the manuscript. A final meeting with all researchers concluded theme development. Text extracts were selected by CS and LG to illustrate the themes and were translated into English to enable publication in an international journal.

Data saturation was not considered in the analytic procedure, as sample selection was pragmatic and saturation has previously been questioned as a part of quality practice in the context of TA [24].

## Results

### Participants

After one participant dropped out due to illness, 39 physicians participated in the focus groups and filled out the survey (see online supplement). Demographic information about the sample is presented in Table 1. The sample was 72% female with an average age of 44 years. Most (62%) were general practitioner specialists, most (64%) worked full-time and most (66%) had worked as physicians for more than five years. When prompted in the survey about the most common precursor to fatigue, most physicians indicated psychosocial stress (78%) and depression (77%). When asked about the intervention they most often recommended, most indicated psychological treatment (82%) or advice on self-care (74%, see the online supplement for full data).

**Table 1:** Demographic information of the physicians who participated in the focus group interviews

		**Total** (*n* = 39)	**PCC1** (*n* = 15)	**PCC2** (*n* = 13)	**PCC3** (*n* = 11)
Gender, n (%)					
	Female	28 (72)	11 (73)	10 (77)	7 (64)
	Male	11 (28)	4 (27)	3 (23)	4 (36)
Age					
	Mean (sd)	45 (11.6)	46 (11.9)	44 (11.6)	46 (12)
Country of birth, n (%)					
	Sweden	28 (72)	11 (73)	10 (77)	7 (64)
	Other	11 (28)	4 (27)	3 (23)	4 (36)
Title, n (%)					
	Non-certified	1 (3)	1 (7)	0	0
	Certified physician	14 (36)	3 (20)	8 (62)	3 (27)
	GP specialist	24 (62)	11 (73)	5 (38)	8 (73)
Years employed in primary care, n (%)					
	< 1 year	6 (15)	1 (7)	3 (23)	2 (18)
	1–5 years	7 (18)	3 (20)	3 (23)	1 (9)
	6–10 years	10 (26)	6 (40)	2 (15)	2 (18)
	> 10 years	16 (41)	5 (33)	5 (38)	6 (55)
Percentage of full-time employment					
	Mean	91	88	94	91

Data on gender and country of birth appear duplicated but has been thoroughly reviewed

*PCC* Primary Care Centre

### Focus group characteristics

The interviews generally began with a sense of tentativeness but became more open and engaged as participants shared their experiences. Many physicians appeared relieved to discover that their colleagues faced similar challenges when treating patients with fatigue. As familiarity and trust grew within the group, participants increasingly built upon each other’s reflections, often reinforcing shared frustrations and offering validation. Rather than engaging in debate, the physicians typically expressed agreement, and moments of silence or hesitation often signalled subtle disagreement rather than direct contradiction.

The moderator facilitated discussion by summarising key points or gently shifting focus when needed, but in most cases, the topics emerged organically, and the interview guide served more as a flexible framework than a strict sequence of questions. Participants who were quieter were occasionally invited to share their thoughts, which often contributed additional nuance or confirmed emerging patterns.

From the opening icebreaker, it became clear that fatigue was associated with feelings of frustration, helplessness, and resignation. These emotions quickly set the tone for the discussions, providing a strong foundation for the analysis of the overarching theme.

### Thematic analysis

#### Overarching theme: Frustration in the role as physician

The overarching theme, *Frustration in the role as physician*, captures a pervasive sense of professional discomfort and emotional strain when managing fatigued patients in primary care.

*“Fatigue and exhaustion disorder, when you see that in the booking you sigh a little to yourself because they are patients that are very difficult to treat. That take time, time we do not have. And I feel that I do not have competence for these patients, you don’t have a lot of treatment to offer.” (PCC 1, interview 1, participant 3)*

This frustration stems not only from clinical uncertainty, limited treatment options, and lack of time, but also from a more profound dissonance between the physicians’ understanding of their professional role and what is demanded of them in these encounters. Physicians described feeling ill-equipped, and in some cases illegitimate, in their role — expressing that they sometimes wondered if they were even doing a doctor’s job.

*“It is a bit challenging to see what your own role in the contact should be and how to spend time and to talk about it [fatigue] - because sometimes it may be that you try to give guidance in areas that do not really strictly belong to the doctor's role.” (PCC 2, interview 1, participant 4)*

The overarching theme encompasses both practical and emotional challenges and is expressed through two main themes. The first, *Time pressure and an empty toolbox*, captures the physicians’ sense of diagnostic and therapeutic inadequacy — where assessments are complex and time-consuming, and treatments often limited or ineffective. The second, *Challenges in the patient–physician relationship*, highlights how encounters with fatigued patients can challenge the physician’s professional identity, particularly when biomedical models fail to explain symptoms, and when psychosocial factors or decisions about sick leave take centre stage. In this theme, physicians grapple with a sense of role conflict, often finding themselves performing tasks — such as offering lifestyle advice, navigating patients’ expectations for sick leave, or negotiating psychological or social factors — that seem to fall outside the traditional boundaries of medical practice.

**Fig. 1:**
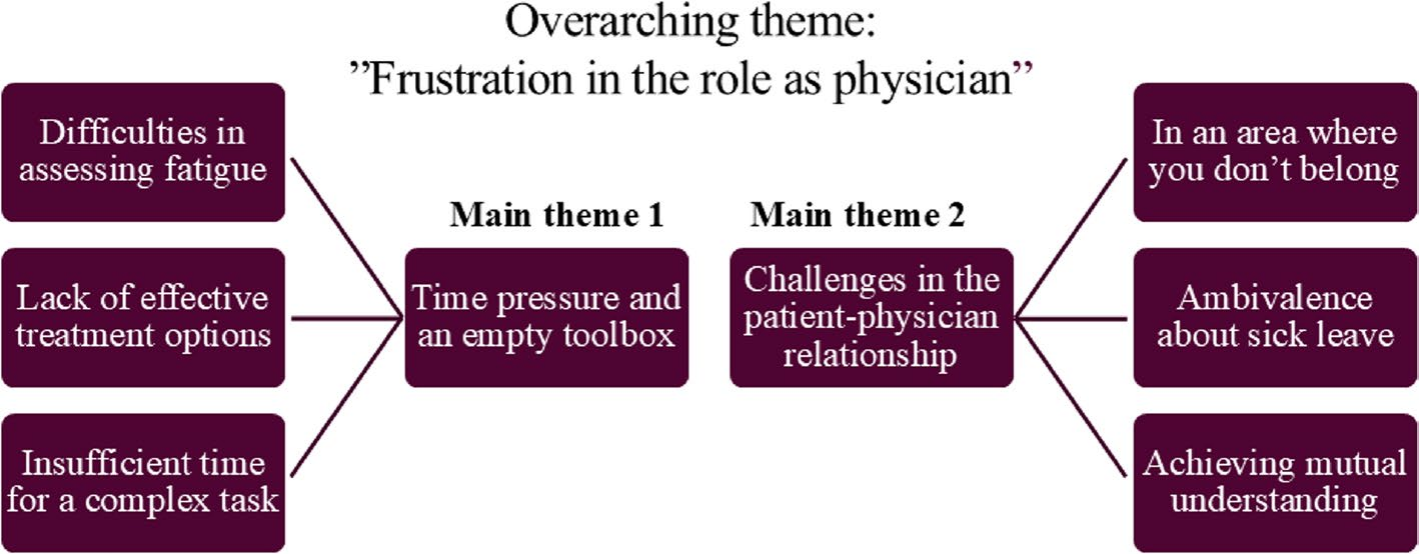
The diagram illustrates the overarching theme of frustration experienced by primary care physicians regarding management of patients with persistent fatigue in primary care. It is divided into two main themes (Theme 1 and Theme 2) that are comprised of subthemes as illustrated in the boxes to the far left and right respectively

Together, these themes describe a complex emotional landscape of frustration, ambivalence, and professional disorientation, in which physicians feel that their ability to fulfil their role is compromised. The following section presents each main theme in detail, along with their subthemes. The thematic map is presented in Fig. 1.

#### Theme 1: Time pressure and an empty toolbox

Physicians are frustrated by the lack of tools available to help patients with fatigue. While the cause of the fatigue can be identified and treated for some patients, discussion centred on the common experience of assessing and treating fatigue when no clear or treatable somatic disorder could be determined. In these cases, the physicians do not feel they can provide patients with either an explanatory diagnosis or an effective treatment.

*“It is a frustration, because as a physician you partly want to find a diagnosis, partly find a treatment that you can offer, and it becomes a frustration when you cannot offer either one”. (PCC3, interview 1, participant 1)*

#### Subtheme 1:1: Difficulties in assessing fatigue

This subtheme captures the frustration of working with fatigue when there are no helpful guidelines or diagnostic labels to guide assessment. Assessment of fatigue requires a substantial effort in working out the cause, progression and current maintaining factors.

*“You have to work out quite a few different things: the purely physical symptoms, could this be something that we need to address? You always investigate a little, like, the social part, work, sleep, alcohol. There are a lot of them. That's also why it's so frustrating. You just imagine that we couldn't do all this in one go [one visit]. After all, it's a patient group where you have to delve into quite a few different areas and not just quickly solve one thing or another…” (PCC2, interview 1, participant 2)*

Many were exasperated by the time-consuming assessment process because they rarely identified any somatic disorder:

*“My first association would be dread and frustration, because it is very hard. It’s very hard to assess. No matter how much I investigate I cannot shake the feeling that there is something I’ve missed. At the same time I have a pretty strong feeling from the beginning that I will probably not find anything” (PCC1, interview 2, participant 1)*

Physicians frequently referred to the Swedish diagnosis of exhaustion disorder as a source of additional frustration which rarely solved the problem of assessment or intervention, and which is difficult to separate from other disorders.

*“We wouldn’t have all these symptom criteria if someone hadn’t invented exhaustion disorder. I don’t know, I’m sorry, I have some pent-up frustration around this.” (PCC3, interview 2, participant 1)*

*“I’ve tried avoiding that diagnosis if you find signs of anxiety or depression. But it might as well have been exhaustion disorder. (PCC2, interview 1, participant 2)*

#### Subtheme 1:2: Lack of effective treatment options

This subtheme captures frustration and resignation due to the lack of treatment options for fatigued patients, and a feeling that even specialist clinics, to which their patients may be referred, cannot provide better treatment.

*“You’ve tried quite a lot of things during a quite long time but there is no effect on anything whatsoever. And then I do not know how to proceed from there.” (PCC2, interview 2, participant 1)*

*“And what is evidence-based? And what might work? What do we do if [cognitive behaviour-therapy] or other treatments do not work? What is the next step? […] otherwise the patient cannot return to work. What do we do then?” (PCC2, interview 1, participant 3)*

Many physicians mentioned specialist rehabilitation clinics offering treatment to patients diagnosed with exhaustion disorder or chronic fatigue syndrome as a source of relief, but questioned their clinical utility:

*“I also think that the specialist clinics do almost nothing. […] They try B12 injections and nothing has an effect […] It’s been a great relief […] to send them to these clinics. […] they have disappeared from us for a while […]. But the way I see it they still come back, most of them, and have about the same plan” (PCC1, interview 1, participant 4)*

#### Subtheme 1:3: Insufficient time for a complex task

Every aspect of the work with fatigued patients is affected by the constant time-pressure that primary care physicians experience, which makes it difficult to conduct a thorough assessment and to discuss factors that could improve the patient’s well-being. With only twenty or thirty minutes per visit, and more than a month between visits, the physicians know they will not be able to adequately address the patient’s problem:

*“I feel I am always lacking time, where I am working extra and doing dictation or journaling very late in my free time … then sometimes being ten minutes late to another patient who is waiting, this time-pressure makes it impossible to explain things or be pedagogical because I do not have time.” (PCC3, interview 2, participant 4)*

Time pressure also contributed to physicians issuing sick leave they otherwise might have avoided, simply because there was no opportunity for deeper discussion about how to help the patient deal with their current situation.

*“We have 30 minutes [...] and then sick leave can be this thing where ‘I am not going to get out of this any other way’, and we say to ourselves that ‘but it will be short’ – but then there is a month-and-a-half queue to see me again.” (PCC1, interview 2, participant 3)*

#### Theme 2: Challenges in the patient-physician relationship

Physicians struggle to manage their fatigued patients when they cannot find any somatic disorder to treat. Many feel that their professional role becomes ambivalent, and challenges in the patient-physician relationship can result in mutual frustration.

*“I think they often feel and think that we do not really understand, because we haven’t been in that same situation, and no one can understand that has not experienced it themselves. … the challenge is not to allow their frustration to spread to you. You try to keep your calm.” (PCC1, interview 1, participant 1)*

This theme also refers to the experience of physicians managing fatigued patients in a way that seems divorced from their traditional role as medical practitioner. Subtheme 2:1: "In an area you don’t belong" in particular explores this tension. Subtheme 2:2 "Ambivalence around sick leave" explores how sick leave has become a central issue of mutual frustration, and in subtheme 2:3: "Achieving mutual understanding", physicians discuss how they try to solve the issue of mutual frustration by becoming more aware of the patient’s view of the problem.

#### Subtheme 2:1: In an area you don’t belong

The physicians repeatedly discussed psychosocial factors (e.g., issues at work, relationship problems, lifestyle factors, or individual psychological factors) as key areas to address with fatigued patients. When dealing with such factors, many physicians felt they no longer provided a medical diagnosis or treatment. This theme thus refers to a complex negotiation with the professional role of the physician, where different physicians reach different conclusions.

*“I thought it was also wise to perhaps not end up in some kind of therapist role, and even if you take the help of other professions that you still somehow step into an area where … [trails off]” (PCC2, interview 1, participant 2)*

“*I try to bring it up as a question that has been somewhat sensitive, but to say like this: ‘Have you thought about switching jobs? It doesn’t seem like you like it there and it’s been like this a long time’. Before, I used to think that it was a bit- as if you were in an area you had no right to be in, but now I think you do.*

*“But the question is, like you said, you are entering an area… that’s not a doctor’s job, it’s more like a guidance counsellor.” (PCC2, interview 1, participant 1 and 4)*

Many physicians feel ambivalent about raising lifestyle issues with the patient. If the patient’s fatigue is related to psychosocial factors, then assessment and treatment, even advice from a physician, risks medicalising the symptoms:

*“One thing that I find challenging is this: what is actually a medical problem and what is not? [Others agree] It becomes a kind of medicalisation to give advice like going out in the woods. [Many agree] It's very much like this: the patient comes to us because they feel unwell, but we can't do anything. This has been a physician’s problem for centuries; we can't fix all problems people experience.” (PCC2, interview 2, participant 3)*

#### Subtheme 2:2: Ambivalence about sick leave

Sick leave emerged as a central source of tension. It was often the patient’s primary expectation and one of the few tangible actions the physician could offer. For many physicians, it was the very first association to “fatigue”:

*“My first thought is this: long sick leave!” [small laugh from the group].” (PCC3, interview 1, participant 3)*

For some it amounted to the chief problem of fatigue:

*“This is where the conflict enters. It’s hard to talk about [fatigue] without talking about the problem of sick leave because if it wasn’t for sick leave there wouldn’t be any problem.” (PCC2, interview 1, participant 1)*

It was discussed as major source of frustration when physician and patient disagreed about the role of sick leave, and some physicians thought that patients took sick leave for granted:

*“I think focus has centred very much on sick leave. […] it’s almost as if they come and say ‘I’m here to write myself sick’. They express it that way. […] You know, you could be making someone passive for tens of years. It’s a very powerful tool that is used too often in my opinion.” (PCC3, interview 2, participant 2)*

Despite it being a common rehabilitative intervention, physicians expressed fear regarding its long-term effects:

*“And if you prescribe sick leave, you are just pouring fuel on the fire.*

*“I agree with you. And it’s exactly this that is the difficulty, to get them back when they’ve been on sick leave.” (PCC2, interview 1, participant 5 and 1)*

#### Subtheme 2:3: Achieving mutual understanding

Many physicians emphasised the importance of aligning with the patient’s understanding of their symptoms and expectations. When such mutual understanding was achieved, it made the encounter feel more manageable and less emotionally draining.

*“If you have a mutual understanding of what the nature of the problem is, then you can always collaborate based on that. […] But if there is no consensus and the patient […] doesn't agree with anything you suggest and perhaps not even on the description of the problem, that's what I would say is the absolute biggest difficulty.*

*“I think ‘mutual understanding‘ is a good umbrella term […] Do we have the same view on how to tackle the problem or not?” (PCC1, interview 2, participant 3 and 1)*

In another interview:

*“But I think it simplifies your work very much and maybe gives less frustration if you know the patient’s expectations. If they are clear that: ‘I want to check I don’t have hypothyroidism, because my mother had it’, then it’s a bounded mission and you don’t have so much performance anxiety”. (PCC3, interview 1, participant 1)*

Mutual understanding thus consists of early identification of the patient’s goal of the consultation, their perception of their illness and their expectations on the physician. Achieving a mutual problem-formulation was understood as particularly important, and challenging, when the symptoms cannot be directly understood from a biomedical perspective.

## Discussion

This study used focus group interviews to explore how primary care physicians in Sweden experience the management of patients with persistent fatigue. The analysis revealed an overarching theme of frustration and inadequacy, rooted not only in clinical uncertainty but also in broader systemic and organisational shortcomings. Physicians described being constrained by a lack of time, standardised guidelines, and effective treatment options — organisational prerequisites that left them feeling ill-equipped to provide safe and effective care. These challenges were compounded by ambivalence about their professional role when no identifiable somatic cause could be found. Taken together, the findings highlight the need to review how fatigue is addressed within the organisation of primary care, clarify the physician’s role in its management, and develop accessible, evidence-based interventions to support both clinicians and patients.

### Previous research

Results from the present study add to an emerging body of qualitative literature suggesting that the management of fatigue and other persistent physical symptoms is generally experienced as demanding and frustrating by healthcare professionals. Previous research on fatigue has primarily focused on individuals with severe fatigue conditions such chronic fatigue syndrome and fibromyalgia where themes of frustration and inadequacy are described as poignant issues for healthcare professionals, together with uncertainty regarding the medical role and the importance of mutual understanding in the physician–patient relationship [25]. The current study is among the few to specifically focus on primary care physicians' perspectives on managing fatigue as a common symptom in primary care [15, 16], and suggests that experiences are similar across healthcare levels. As early identification and effective management of fatigue has been argued to be crucial in preventing long-term disability and healthcare consumption [16], increased attention to the primary care context appears warranted.

### Concerns about exhaustion disorder

In the Swedish healthcare context, the exhaustion disorder diagnosis is routinely used in primary care to signify clinically impairing fatigue that is assumed to be caused by long-term stress. One might expect that the availability of this diagnosis would improve management of fatigue by providing physicians with a clear diagnostic category. However, our results suggest that physicians have mixed opinions about the clinical utility of the diagnosis, and that many refrain from using it, likely due to the lack of evidence-based interventions available to treat patients with exhaustion disorder [11].

### Fatigue and the medical role

In line with previous research, we found that a recurring theme among physicians is the uncertainty surrounding the role of healthcare in supporting patients with fatigue that is not accounted for by identifiable somatic disorder [15, 16, 25]. Many physicians in this study expressed uneasiness about advising patients on aspects of fatigue that they thought were relevant for the patient but outside the traditional purview of medicine, fearing it will shift responsibility for managing stressful life events to healthcare services. At the same time, many patients consider it a key medical outcome to understand their condition when they seek healthcare [8], reflecting a need for healthcare workers to be able to deliver a rational explanation for the symptoms. A recent randomised trial found that physicians trained in communication support for persistent physical symptoms helped reduce patients’ symptom severity, healthcare use, and sickness absence [26]. This suggests that improving physicians' communication skills about fatigue can enhance patient outcomes while minimising unnecessary healthcare.

Fatigue thus poses challenges to many physicians, who do not have time, feel competent, and/or do not see it as their primary role to address this multifaceted symptom. There is a pressing need to develop guidelines to support physicians in their assessment and management of this patient group. Recent advances in the understanding of fatigue and other persistent physical symptoms as multifactorial phenomena [4] must be effectively communicated to primary care physicians to empower them in their clinical decision-making. Our findings suggest that such interventions would be well-received by primary care physicians, who repeatedly expressed the need to improve their competence in managing fatigue. These measures could also help reduce the role ambivalence reported by the physicians in this study.

### Strengths and limitations

This study has several strengths. The use of focus groups fostered natural discussion and provided a nuanced understanding of physicians’ experiences. Furthermore, the inclusion of multiple primary care centers provided a broad perspective on physicians’ experiences, and a relatively large number of physicians were included.

Limitations include a sample limited to three primary care centers in Stockholm, which may not capture the full diversity of experiences across different regions. The study did not include perspectives from other healthcare professionals, such as nurses or mental health specialists, who may have other perspectives on managing fatigue. Unlike a previous study on fatigue in primary care that incorporated both patient and physician perspectives [16], our study did not include patient viewpoints. Including these may have provided additional insight into the mutual frustration frequently described by physicians in the interviews.

## Conclusions

Persistent fatigue entails significant challenges for primary care physicians, who report feeling frustrated, ill-equipped, and constrained by time when addressing this multifaceted and disabling symptom. These experiences are consistent with previous qualitative studies, highlighting a broader issue of healthcare professionals grappling with their role in addressing fatigue, especially when it is not linked to a discernible somatic cause. There is a pressing need to improve the organisational prerequisites for physicians managing patients with fatigue and to develop accessible, effective, evidence-based treatments adapted for use in the primary care context.

## Supplementary Information

Supplementary Material 1.

## Data Availability

The study was pre-registered on Open Science Framework (DOI: 10.17605/OSF.IO/C9YBQ). The datasets generated and analysed during the current study are not publicly available in order to protect the integrity of the participants, but are available from the corresponding author on reasonable request.

## Abbreviations

PCC: Primary Care Centre
TA: Thematic Analysis
