# Supplementary material for: Physicians’ experiences of assessing and supporting fatigued patients in primary care: a focus group study
Source: BMC Prim Care. 2025 Jun 9;26:197. doi: 10.1186/s12875-025-02891-1 (PMC12147317; doi:10.1186/s12875-025-02891-1)
Supplement: Supplementary file 1 — Supplementary Material 1. [file 12875_2025_2891_MOESM1_ESM.docx]

Online Supplementary Material

Physicians’ experiences of assessing and supporting fatigued patients in primary Care: A focus group study

Conrad Samuelsson^1,2^, Lisa Gunnarsson ^3^, Frank Svärdman^1,2^, Christian Rück ^3^, Elin Lindsäter ^1-3^.

Affiliations

1. Gustavsberg University Primary Healthcare Clinic, Academic Primary Care Center, Region Stockholm, Stockholm, Sweden
2. Division of Psychology, Department of Clinical Neuroscience, Karolinska Institutet, Stockholm, Sweden
3. Centre for Psychiatry Research, Department of Clinical Neuroscience, Karolinska Institutet, & Stockholm Health Care Services, Region Stockholm, Stockholm, Sweden

Corresponding author: Conrad Samuelsson ([conrad.samuelsson@ki.se](mailto:conrad.samuelsson@ki.se))

Table of contents

[1. Survey questions 1](#_Toc194321815)

[Background Questions 1](#_Toc194321816)

[Tiredness 2](#_Toc194321817)

[2. Survey responses 3](#_Toc194321818)

[3. Interview protocol 4](#_Toc194321819)

# 1. Survey questions

## Background Questions

1. **Gender**

a. Female
b. Male
c. Other

1. **Year of Birth**: __________
2. **Country of Birth**
   a. Sweden
   b. Other
3. **Title**
   a. Unlicensed physician
   b. Licensed physician
   c. Specialist in general medicine
4. **Number of years employed in primary care**
   a. <1 year
   b. 1-5 years
   c. 6-10 years
   d. >10 years
5. **What percentage of full-time work do you work at this primary care centre?**
   a. 25%
   b. 50%
   c. 75%
   d. 100%
   e. Other (Specify: ________________)

## Tiredness

The following three questions concern long-term (more than 3 months), functionally impairing tiredness that does not improve with rest and where you have ruled out somatic explanations for why the tiredness continues.

1. **Approximately what percentage of your patients report feeling such tiredness?**
   a. <10%
   b. 10–20%
   c. 20–30%
   d. 30–40%
   e. 40–50%
   f. >50%
2. **In your experience, which of the following most often precedes such tiredness?**
   a. Endocrine disease
   b. Neurological disease
   c. Gastrointestinal disease
   d. Hematological disease
   e. Infection
   f. Cardiovascular disease
   g. Musculoskeletal disease
   h. Cancer
   i. Depression
   j. Anxiety disorder
   k. Insomnia
   l. Neuropsychiatric disorder
   m. Personality disorder
   n. Side effects of pharmacological treatment
   o. Social (e.g., related to problems in work life, relationships, finances)
   p. Other (Specify: ______________________________________________________)
3. **What are the most common measures you, as a physician, take to help a patient suffering from such fatigue?**
   a. Advice on self-care
   b. Physical activity on prescription
   c. Pharmacological treatment (If yes, specify type ___________________________)
   d. Supportive conversations with the patient
   e. Sick leave
   f. Psychological assessment/treatment in primary care
   g. Physiotherapy/rehabilitation in primary care
   h. Referral to specialist clinic for further investigation
   i. Referral to rehabilitation clinic
   j. Other (Specify: _____________________________________________________)

Thank you very much for your participation!

# 2. Survey responses

|  |  | Total  (n = 39) | PCC1  (n = 15) | PCC2  (n = 13) | PCC3  (n = 11) |
| --- | --- | --- | --- | --- | --- |
| What proportion of patients present with fatigue* each week? n (%) | | | | | |
|  | <10% | 21 (54) | 8 (53) | 8 (62) | 5 (45) |
|  | 10–20% | 8 (21) | 4 (27) | 1 (8) | 3 (27) |
|  | 20–30% | 4 (10) | 2 (13) | 2 (15) | 0 |
|  | 30–40% | 4 (10) | 0 | 1 (8) | 3 (27) |
|  | 40–50% | 0 | 0 | 0 | 0 |
|  | >50% | 0 | 0 | 0 | 0 |
|  | no reply | 2 (5) | 1 (7) | 1 (8) | 0 |
| In your experience, what factors usually precede fatigue? n (%) | | | | | |
|  | Psychosocial problems | 31 (79) | 14 (92) | 12 (92) | 6 (55) |
|  | Depression | 30 (77) | 10 (67) | 10 (77) | 10 (91) |
|  | Anxiety | 27 (69) | 9 (69) | 11 (85) | 7 (64) |
|  | Insomnia | 19 (49) | 7 (47) | 5 (38) | 7 (64) |
|  | Infection | 16 (41) | 5 (33) | 6 (46) | 5 (45) |
|  | Personality traits | 16 (41) | 6 (40) | 6 (46) | 4 (36) |
|  | Neuropsychiatric disorder | 15 (38) | 6 (40) | 7 (54) | 4 (36) |
|  | Cardiovascular disorders | 10 (26) | 4 (27) | 4 (31) | 2 (18) |
|  | Musculoskeletal disorders | 8 (21) | 2 (13) | 5 (38) | 1 (9) |
|  | Cancer | 7 (18) | 2 (13) | 4 (31) | 1 (9) |
|  | Endocrine disorders | 7 (18) | 3 (20) | 2 (15) | 2 (18) |
|  | Neurological disorders | 7 (18) | 3 (20) | 4 (31) | 0 (0) |
|  | Hematological disorders | 2 (5) | 1 (7) | 0 (0) | 1 (9) |
|  | Pharmacological | 2 (5) | 1 (7) | 1 (8) | 0 (0) |
|  | Gastrointestinal disorders | 1 (3) | 0 (0) | 1 (8) | 0 (0) |
| What interventions do you usually recommend to fatigued patients? n (%) | | | | | |
|  | Psychological treatment | 32 (82) | 14 (93) | 10 (77) | 8 (73) |
|  | Advice on self-care | 29 (74) | 12 (80) | 8 (62) | 9 (82) |
|  | Physiotherapy | 23 (59) | 7 (47) | 9 (69) | 7 (64) |
|  | Sick leave | 17 (44) | 7 (47) | 9 (69) | 7 (64) |
|  | General support | 14 (36) | 6 (40) | 5 (38) | 3 (27) |
|  | Pharmacological | 13 (33) | 6 (40) | 2 (15) | 5 (45) |
|  | Specialist rehabilitation | 12 (31) | 7 (47) | 3 (23) | 2 (18) |
|  | Specialist assessment | 10 (26) | 8 (53) | 1 (8) | 1 (9) |
|  | Physical activity | 9 (23) | 0 (0) | 3 (23) | 6 (55) |
| *Note:* PCC = Primary Care Centre.  * Defined in the survey as “severe, persistent tiredness not alleviated by rest and not explained by an identifiable disease process”. | | | | | |

# 3. Interview protocol

**Introduction**

Tiredness is one of the most common symptoms in primary care, and can arise as a consequence of a multitude of causes, including infections, iron deficiencies, head trauma, etc. Sometimes the tiredness is completely without somatic explanation, or the somatic cause of the tiredness is already well treated. For example, if the tiredness came as a symptom of an infection, but persists even when the infection is treated.

When tiredness persists for a long time and leads to disability we sometimes refer to it as “chronic tiredness”, “fatigue”, or “exhaustion”. As primary care physicians you are generally the first line of treatment for these patients. The goal of this focus group is for us to understand more about how you manage patients with persistent and disabling tiredness in primary care.

Our research group is working to increase knowledge about the clinical picture of persistent tiredness, and we want to investigate if there could be common maintaining factors across patient groups. This focus group study is a preparatory study to a clinical treatment study where we want to study the effect of transdiagnostic psychological treatment of severe tiredness in primary care.

We hope to get to hear your unfiltered thoughts and perspectives around persistent tiredness, what challenges you face in your everyday clinical practice and what you think about possible interventions to reduce tiredness.

**Questions**

(1) What are your first associations to severe tiredness, or "fatigue"?

a. Do you get different associations depending on whether the term tiredness, exhaustion, or fatigue is used?

(2) What questions are important to ask patients who express severe fatigue to understand the clinical picture and suggest an intervention?

(3) In cases where there is no clear medical explanation for the fatigue,

a. How do you communicate this to the patient?

b. What diagnostic codes are available?

c. What interventions do you usually recommend?

(4) How do you feel about meeting patients with severe long-term fatigue?

a. How satisfied are you with the available methods for assessing and diagnosing severe fatigue?

b. What are the biggest challenges you encounter when it comes to assessing and diagnosing severe fatigue?

c. Do you have any thoughts on what could help the work of assessing and diagnosing severe fatigue?

**Concluding**

Thank you for participating today, your responses to these questions are instrumental to improving future care for this patient group.
